# Revised evaluation objectives of the Korean Dentist Clinical Skill Test: a survey study and focus group interviews

**DOI:** 10.3352/jeehp.2024.21.11

**Published:** 2024-05-30

**Authors:** Jae-Hoon Kim, Young J Kim, Deuk-Sang Ma, Se-Hee Park, Ahran Pae, June-Sung Shim, Il-Hyung Yang, Ui-Won Jung, Byung-Joon Choi, Yang-Hyun Chun

**Affiliations:** 1 Department of Dental Education, Dental and Life Science Institute, School of Dentistry, Pusan National University and Dental Research Institute, Yangsan, Korea; 2 Seoul National University School of Dentistry, Seoul, Korea; 3 Department of Preventive and Public Health Dentistry, College of Dentistry, Gangneung-Wonju National University & Research Institute of Oral Science, Gangneung, Korea; 4 Department of Conservative Dentistry, Gangneung-Wonju National University, Gangneung, Korea; 5 Department of Prosthodontics, College of Dentistry, Kyung Hee University, Seoul, Korea; 6 Department of Prosthodontics, Yonsei University College of Dentistry, Seoul, Korea; 7 Department of Orthodontics, Seoul National University School of Dentistry, Seoul, Korea; 8 Department of Periodontology, Research Institute for Periodontal Regeneration, Yonsei University College of Dentistry, Seoul, Korea; 9 Department of Oral and Maxillofacial Surgery, Kyung Hee University, Seoul, Korea; 10 Department of Oral Medicine, Kyung Hee University, Seoul, Korea; Hallym University, Korea

**Keywords:** Clinical competence, Dentists, Focus groups, Professional role, Republic of Korea

## Abstract

**Purpose:**

This study aimed to propose a revision of the evaluation objectives of the Korean Dentist Clinical Skill Test by analyzing the opinions of those involved in the examination after a review of those objectives.

**Methods:**

The clinical skill test objectives were reviewed based on the national-level dental practitioner competencies, dental school educational competencies, and the third dental practitioner job analysis. Current and former examinees were surveyed about their perceptions of the evaluation objectives. The validity of 22 evaluation objectives and overlapping perceptions based on area of specialty were surveyed on a 5-point Likert scale by professors who participated in the clinical skill test and dental school faculty members. Additionally, focus group interviews were conducted with experts on the examination.

**Results:**

It was necessary to consider including competency assessments for “emergency rescue skills” and “planning and performing prosthetic treatment.” There were no significant differences between current and former examinees in their perceptions of the clinical skill test’s objectives. The professors who participated in the examination and dental school faculty members recognized that most of the objectives were valid. However, some responses stated that “oromaxillofacial cranial nerve examination,” “temporomandibular disorder palpation test,” and “space management for primary and mixed dentition” were unfeasible evaluation objectives and overlapped with dental specialty areas.

**Conclusion:**

When revising the Korean Dentist Clinical Skill Test’s objectives, it is advisable to consider incorporating competency assessments related to “emergency rescue skills” and “planning and performing prosthetic treatment.”

## Graphical abstract

[Fig f2-jeehp-21-11]

## Introduction

### Background/rationale

In 2009, the Korean Dental Association decided to introduce a clinical skill test as part of the Korean Dental Licensing Examination (KDLE). The objectives of the clinical skill test were developed to assess the minimal competencies that new dentists should possess, based on dental practitioners’ competencies in conjunction with the dental practitioner job analysis. In 2011, national-level dental practitioner competencies were developed to establish evaluation objectives for the Korean Dentist Clinical Skill Test [1]. The Korean Health Personnel Licensing Examination Institute (KHPLEI) conducted the first dental practitioner job analysis in 2000 and the second dental practitioner job analysis in 2012 [2]. In 2013, a study was conducted on the categories and item types of the clinical skill test, revising the selection criteria of the test’s items within the national-level dental practitioner competencies, which were developed in 2011 (Supplement 1) [3]. The researchers extracted “task elements” that could be tested in the clinical skill test from the second dental practitioner job analysis and correlated 36 extracted task elements with the national-level dental practitioner competencies. Subsequently, the affairs committee determined the clinical skill test’s assessment categories and types of test items.

In 2017, KHPLEI published evaluation objectives for the Korean Dentist Clinical Skill Test, which currently consists of process and outcome evaluations (Table 1). The “process evaluation” is administered in the form of an objective structured clinical skill examination, which includes a combination of one or more of the 9 domains of assessment [4]. The “outcome evaluation” is a form of bench test using a dental mannequin simulator. The evaluation objectives list 22 items to be evaluated (Table 2).

The first clinical skill test took place in 2021, and 3 examinations were conducted by the end of 2023. In 2023, the third dental practitioner job analysis was published [5]. Given that the competencies necessary for dentists, dental education, and the practice environment are continually evolving, it is imperative to periodically review the evaluation objectives of the Korean Dentist Clinical Skill Test.

### Objectives

The study aimed to propose a plan for revising the Korean Dentist Clinical Skill Test based on the opinions of those involved in the examination after reviewing the evaluation objectives based on the national-level dental practitioner competencies, dental school educational competencies, and the third dental practitioner job analysis.

## Methods

### Ethics statement

This study was approved by the Institutional Review Board (IRB) of Kyung Hee University Dental Hospital (IRB no., KH-DT23032).

### Study design

This was a survey-based study with focus group interviews. The literature review on the clinical skill test objectives was based on the national-level dental practitioner competencies, dental school educational competencies, and the third dental practitioner job analysis.

### Setting

#### Reviewing the validity of the clinical skill test’s evaluation objectives

**Fig. 1.**
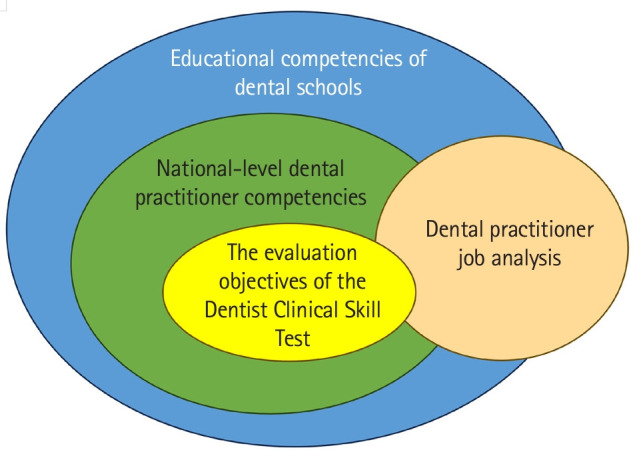
Study design for validating the evaluation objectives of the Korean Dental Clinical Skill Test.

The design for examining the validity of the evaluation objectives of the Korean Dentist Clinical Skill Test is presented in Fig. 1. The evaluation objectives were reviewed based on the national-level dental practitioner competencies and the educational competencies of dental schools across the country. Next, the examination objectives were reviewed based on the results of the third dental practitioner job analysis.

Educational competency data from 11 dental schools nationwide were collected. From the educational competency data and the national-level dental practitioner competencies, “competency elements” relevant to the 22 clinical skill test objectives were extracted. The authors discussed the validity of the competency elements as clinical skill test objectives and the feasibility of test item development for those competency elements.

The third dental practitioner job analysis published in 2023 was based on the second dental practitioner job analysis [5]. In the third dental practitioner job analysis, dental practitioners working in private dental clinics with at least 5 years of practical experience were asked to rate their perceptions of task importance using a 5-point Likert scale (1, not important: 2, somewhat important; 3, moderately important: 4 points, important: 5, extremely important). In this study, task elements related to dentist competency with an average importance rating of 4 or higher were selected. The authors assessed the feasibility and ease of implementing the task elements as examination objectives.

#### Perceptions of the clinical skill test’s evaluation objectives

Surveys were conducted to gather perspectives from individuals involved in the examination, including current and former examinees, professors participating in the examination, and dental school faculty members, regarding their perceptions of the evaluation objectives.

### Participants

The examinees were students who attempted the 76th KDLE in 2023, and the former examinees were licensed dentists who had taken the KDLE in 2021 and 2022. The participating professors were those who had participated in the examination as either test item developers or test examiners. The dental school faculty members were affiliated with the 11 dental schools in Korea.

### Data sources/measurement

Three surveys were conducted to gather data for this study. The first survey targeted current and former examinees, assessing their perceptions of the evaluation objectives and the alignment of these objectives with their school curricula (Supplement 2). The second and third surveys were administered to the participating professors and dental school faculty members, respectively, focusing on their perceptions of the validity of the 22 evaluation objectives (Supplements 3, 4). For the participating professors (test item developers and test examiners), an additional item on the overlap between the 22 evaluation objectives and the dental specialty areas was included (Supplement 3). All surveys employed a 5-point Likert scale.

### Bias

All participated in the survey voluntarily. There may have been selection bias because individuals with a positive attitude toward the clinical skill test may have participated in the survey more actively.

### Study size

The sample size was not predetermined, as all participants took part voluntarily.

### Statistical methods

The survey responses were subjected to descriptive analysis. The chi-square test was used to compare responses between the groups. All statistical analyses were performed using IBM SPSS ver. 25.0 (IBM Corp.), and the statistical significance level was set at α=0.05.

### Focus group interviews with clinical skill test experts

Two focus group interviews were conducted with experts with extensive experience participating in the clinical skill test for the process and outcome evaluations. During the focus group interviews, participants discussed which evaluation objectives should be retained, deleted, revised, or added. Each focus group interview included 5 clinical skill test experts.

## Results

### Reviewing the validity of the clinical skill test’s evaluation objectives

When the extracted competency elements were compared to the evaluation objectives, it was found that some competency elements were not included in the clinical skill test. These competency elements were “periodontal curettage,” “supportive periodontal therapy,” “diagnosis and treatment planning–simple soft tissue diseases,” “incision and drainage,” “biopsy,” “treatment of oromaxillofacial pain,” and “emergency rescue skills.” The results of our review on the feasibility and ease of implementing these competency elements as clinical skill tests are presented in Table 3. Among the task elements identified in the third dental practitioner job analysis, those related to dentist competency that had an average importance rating of 4 or higher were selected and assessed for alignment with the evaluation objectives. The results of the validity assessment and the feasibility of incorporating these task elements as evaluation objectives are presented in Table 4.

### Survey perceptions of the clinical skill test’s evaluation objectives

In total, 116 current and 35 former examinees completed the survey, and the results are available in Dataset 1 and summarized in Table 5. No statistically significant differences were found between the perceptions of the current and former examinees.

The survey on the perceptions of the validity of clinical skill test objectives included responses from 16 test item developers, 32 test examiners, and 24 dental school faculty members. The results of the survey are available in Dataset 2 and summarized in Table 6. For most items, the percentages of “agree” and “strongly agree” were high, with no significant differences between the groups. There were differences between groups on some items, such as “4. Pulp test,” where the percentage of “strongly agree” was relatively high among dental school professors. For “15. Space management for primary and mixed dentition,” the percentage of test item developers who strongly disagreed was relatively high.

The responses of the participating professors to the survey item “Do the evaluation objectives overlap with dental specialty areas?” are available in Dataset 3 and summarized in Table 7. There were no significant differences between the groups on any of the items. Most of the items had a high percentage of “disagree” or “strongly disagree” responses, implying that the evaluation objectives do not overlap with the dental specialty areas. However, “2. Oromaxillofacial cranial nerve examination,” “3. Temporomandibular disorder palpation test,” and “15. Space management for primary and mixed dentition” were more likely to be perceived as overlapping with dental specialty areas than other items.

### Focus group interviews with clinical skill test experts

Clinical skill test experts agreed that most of the evaluation objectives should be retained considering the competencies required for new dentists. However, in the focus group interview for the “process evaluation,” some participants suggested that “2. Oromaxillofacial cranial nerve examination” and “15. Space management for primary and mixed dentition” were inappropriate for evaluating the competencies of new dentists. Regarding the objectives that require revision, “6. Intraoral X-ray taking” was discussed due to practical difficulties in exam administration. In the focus group interview for the “outcomes evaluation,” the experts suggested that the material-specific evaluation objectives should be revised to include various restorative and prosthetic materials. Additionally, several experts proposed adding items related to dental laboratory procedures, preparation of abutment teeth for removable partial dentures, and pediatric dentistry to the evaluation objectives.

## Discussion

### Key results

In this study, the evaluation objectives of the Korean Dentist Clinical Skill Test were reviewed based on the national-level dental practitioner competencies, dental school curricula, and the third dental practitioner job analysis. The evaluation objectives for the Korean Dentist Clinical Skill Test were developed in conjunction with the development of dental practitioners’ competencies. Dental schools in Korea are working toward implementing performance-based education based on the national-level dental practitioner competencies. This study confirmed that the clinical skill test’s evaluation objectives encompass most of the basic competencies expected of dentists graduating from dental schools. However, it was found that some competencies required of new dentists, such as “emergency rescue skills,” “planning and performing simple fixed partial denture treatment,” and “planning and performing simple removable partial denture treatment,” were not included in the evaluation objectives.

### Interpretation

The survey respondents recognized that a majority of the clinical skill test’s objectives were appropriate to assess the competence of new dentists. However, some evaluation objectives needed reviewing given the environment in which the clinical skill test is administered and considering some objective overlap with dental specialty areas.

Regarding “2. Oromaxillofacial cranial nerve examination,” numerous opinions from professors participating in the examination and from dental schools indicated that it was inadequate as an evaluation objective. This is likely because they believed that the topic falls under a specialty area. Moreover, in the job analysis data, the cranial nerve exam was rated low in importance and frequency.

For professors participating in the clinical skill test, many respondents stated that “3. Temporomandibular disorder palpation test” falls under a specialty area. The focus group interviews also indicated that this item was difficult to adequately assess via the clinical skill test.

Many dental school faculty members considered “6. Intraoral X-ray taking” to be an appropriate evaluation objective, whereas professors participating in the examination felt that it was inappropriate. These differing views may stem from the recognition that competency in intraoral radiography is essential for new dentists, but it can be challenging to access radiographic equipment within the constraints of the current clinical skill test environment. The focus group interviews suggested that competency should be assessed by other means, such as multimedia questions, which are being considered for inclusion in the KDLE.

Many professors involved in the clinical skill test agreed that “15. Space management for primary and mixed dentition” was unsuitable as an evaluation objective. This may be due to the perception that this area is specialized and the challenges of creating assessment questions.

The percentage of participants who agreed that “18. Amalgam restoration in posterior teeth” was valid as a clinical skill test objective was relatively lower than that for other evaluation objectives. In the focus group interview, participants recommended either removing or revising the objective related to amalgam restorations, noting that amalgam is now seldom used. It was suggested that the evaluation objective should be revised to “intracanal restorations” to include a range of restorative materials including composite resins rather than limiting the evaluation objective to amalgam.

### Limitations

The limitations of this study include its focus on reviewing the clinical skill test’s evaluation objectives based primarily on the national-level dental practitioner competencies, dental school curricula, and dentist job analysis—all of which are specific to Korea. Future studies should also consider the implementation and evolution of clinical skill tests in other countries to provide a more comprehensive perspective on potential improvements for the KDLE.

### Suggestions

Emergency rescue skills, which constitute a competency directly related to patient life, are included as an evaluation objective in national examinations for other healthcare professions, such as physicians and emergency medical technicians. This item is related to “7.3. Perform emergency medical procedures that may occur in a dental practice” in the national-level dental practitioner competencies. The dental practitioner job analysis includes “B3-6 Perform cardiopulmonary resuscitation,” which was identified as a low-frequency but high-importance item. Given that the primary purpose of the KDLE is to assess minimal competency as a healthcare provider, it is reasonable to consider adding test items related to emergency rescue skills, which can be evaluated using a first aid simulator.

“Planning and performing simple fixed partial denture treatment” and “planning and performing simple removable partial denture treatment” are among the important competencies for dentists, relating to “6.19. Can effectively design and perform simple fixed and removable partial denture, and full denture treatments” in the national-level dental practitioner competencies and “B4-6 Perform a fixed partial denture treatment” and “B4-8 Perform a removable partial denture treatment” in the dental practitioner job analysis. These competencies cover a wide range of clinical skill-related situations and competency levels. Therefore, a comprehensive discussion should be held on the scope and level of these competencies. Regarding the evaluation method, considering that the competencies related to prosthetic treatments are currently evaluated by the “outcome evaluation” using a dental mannequin simulator, it could be possible to evaluate “planning and performing simple fixed partial denture treatment” and “planning and performing simple removable partial denture treatment” via the “outcome evaluation.”

### Conclusion

Based on an analysis of the national-level dental practitioner competencies, dental school educational competencies, dental practitioner job analysis, and the perspectives of those involved in the clinical skill test, this study found that most evaluation objectives were valid. However, future revisions should incorporate assessments related to “emergency rescue skills” and “planning and performing prosthetic treatment.” Modifications to the evaluation objectives of the Korean Dentist Clinical Skill Test should involve input from both dental education institutions and specialty societies to achieve a collective consensus among dental professionals.

## Figures and Tables

**Figure f2-jeehp-21-11:**
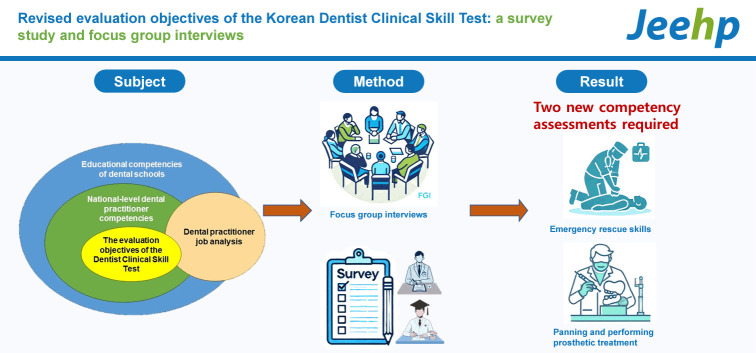


**Table 1.** Item types and evaluation areas of the Korean Dentist Clinical Skill Test

Item types	Evaluation areas
Process evaluation	(1) Patient interview
	(2) Intraoral examination
	(3) Extraoral examination
	(4) Radiographic examination
	(5) Treatment planning
	(6) Preventive treatment
	(7) Management of malocclusion
	(8) Periodontal and oral soft tissue treatment
	(9) Surgical treatment
Outcome evaluation	(1) Operative treatment
	(2) Endodontic treatment
	(3) Prosthodontic treatment

**Table 2.** The evaluation objectives of the Korean Dentist Clinical Skill Test

Evaluation objectives (22 items)
1. Patient interview, history taking, diagnosis, and treatment planning
2. Oromaxillofacial cranial nerve examination
3. Temporomandibular disorder palpation test
4. Pulp test
5. Periodontal test
6. Intraoral X-ray taking
7. Local anesthesia
8. Rubber dam application
9. Topical fluoride application
10. Pit and fissure sealant
11. Scaling
12. Root planning
13. Simple extraction
14. Suturing skills
15. Space management for primary and mixed dentition
16. Oral hygiene education
17. Endodontic treatment
18. Amalgam restoration in posterior teeth
19. Resin composite restoration in anterior teeth
20. Gold inlay cavity preparation in posterior teeth
21. Gold crown preparation in posterior teeth and fabrication of a temporary crown
22. Porcelain fused to metal crown in anterior teeth and fabrication of a temporary crown

**Table 3.** Competency elements not included in the clinical skill test objectives

Competency element	National-level dental practitioner competencies	Validity (+/-)	Feasibility of item development (+/-)
Periodontal curettage	6.8. Perform periodontal curettage for chronic periodontitis.	+	-
Supportive periodontal therapy	6.9. Perform supportive periodontal treatment.	-	-
Diagnosis and treatment plan-simple soft tissue diseases	6.10. Treat simple soft tissue conditions.	+	+
Incision and drainage	6.16. Perform simple incision and drainage.	+	-
Biopsy	6.17. Perform simple tissue excision for biopsy.	+	-
Planning and performing prosthetic treatment	6.19. Effectively design and perform simple fixed and removable partial dentures, and full dentures.	+	+
Treatment of oromaxillofacial pain	6.23. Treat oral and maxillofacial pain, including temporomandibular joint.	+	+
Emergency rescue skills	7.3. Perform first aid procedures that may occur in a dental office.	+	+

**Table 4.** Task elements not included in the clinical skill test objectives

Task	Task frequency^a)^	Task importance^b)^	Competency element	Validity (+/-)	Feasibility of item development (+/-)
B1. Conservative treatment					
B1-5. Pulp capping	1.72	4.19	Diagnosis and treatment planning	-	-
B1-6. Pulpectomy	1.74	4.08	Diagnosis and treatment planning	-	-
B2. Periodontal and oral soft tissue treatment					
B2-4. Crown lengthening	2.02	4.32	Simple surgical treatment	+	-
B2-5. Gigivectomy	2.09	4.09	Simple surgical procedures	+	-
B3. Surgical treatment					
B3-3. Extraction of impacted teeth	2.45	4.45	Simple surgical extraction	+	-
B3-4. Incision and drainage	2.34	4.58	Incision and drainage	+	-
B3-6. Cardiopulmonary resuscitation	1.00	4.17	Emergency rescue skills	+	+
B3-11. Implant placement	2.83	4.81	Planning and performing prosthetic treatment	-	-
B4. Prosthetic treatment					
B4-7. Implant prosthesis	2.85	4.79	Planning and performing prosthetic treatment	-	-
B4-5. Prefabricated crown treatment	1.87	4.17	Treatment of pediatric and adolescent patients	-	+
B4-6. Fixed partial denture treatment	2.02	4.40	Planning and performing prosthetic treatment	+	+
B4-8. Removable partial denture treatment	1.94	4.53	Planning and performing prosthetic treatment	-	+
B4-9. Full denture treatment	1.94	4.53	Planning and performing prosthetic treatment	-	-
B4-11. Post and core	2.34	4.57	Extra-coronal restorations	+	-

a) 1, no more than once per year; 2, at least once per month; 3, at least once per week; 4, at least once per day.

b) 1, not important; 2, somewhat important; 3, moderately important; 4, important; 5, extremely important.

**Table 5.** Survey results of current and former examinees regarding the evaluation objectives of the clinical skill test

Item	Response (no., %)					P-value^a)^
	Strongly disagree	Disagree	Neutral	Agree	Strongly agree	
Do you know the evaluation objectives for the Korean Dentist Clinical Skill Test?						0.405
Examinees (n=116)	2 (1.7)	5 (4.3)	20 (17.2)	47 (40.5)	42 (36.2)	
Former examinees (n=35)	0	2 (5.7)	11 (31.4)	12 (34.3)	10 (28.6)	
Have you taken any preparation training for the examination offered by your school?						0.451
Examinees (n=116)	1 (0.9)	2 (1.7)	16 (13.8)	45 (38.8)	52 (44.8)	
Former examinees (n=35)	0	2 (5.7)	2 (5.7)	15 (42.9)	16 (45.7)	
Did the evaluation objectives help you prepare for the examination?						0.517
Examinees (n=116)	4 (3.4)	8 (6.9)	29 (25.0)	48 (41.4)	27 (23.3)	
Former examinees (n=35)	0	0	9 (25.7)	17 (48.6)	9 (25.7)	
Did the evaluation objectives help you develop basic competencies as a dentist?						0.183
Examinees (n=116)	3 (2.6)	11 (9.5)	24 (20.7)	53 (45.7)	25 (21.6)	
Former examinees (n=35)	0	0	9 (25.7)	21 (60.0)	5 (14.3)	
Do you think the “process evaluation” objectives reflect the competencies required of new dentists?						0.345
Examinees (n=116)	8 (6.9)	10 (8.6)	29 (25.0)	46 (39.7)	23 (19.8)	
Former examinees (n=35)	0	1 (2.9)	9 (25.7)	19 (54.3)	6 (17.1)	
Do you think the “process evaluation” objectives reflect the educational content of your school?						0.674
Examinees (n=116)	5 (4.3)	6 (5.2)	30 (25.9)	53 (45.7)	22 (19.0)	
Former examinees (n=35)	1 (2.9)	2 (5.7)	11 (31.4)	18 (51.4)	3 (8.6)	
Do you think the test items of the “process evaluation” are aligned with the evaluation objectives?						0.430
Examinees (n=116)	5 (4.3)	5 (4.3)	32 (27.6)	55 (47.4)	19 (16.4)	
Former examinees (n=35)	0	3 (8.6)	7 (20.0)	21 (60.0)	4 (11.4)	
Do you think the “outcome evaluation” objectives reflect the competencies required of new dentists?						0.089
Examinees (n = 116)	2 (1.7)	5 (4.3)	19 (16.4)	53 (45.7)	37 (31.9)	
Former examinees (n = 35)	0	1 (2.9)	7 (20.0)	19 (54.3)	8 (22.9)	
Do you think the “outcome evaluation” objectives reflect the educational content of your school?						0.600
Examinees (n=116)	2 (1.7)	2 (1.7)	19 (16.4)	62 (53.4)	31 (26.7)	
Former examinees (n=35)	0	1 (2.9)	9 (25.7)	18 (51.4)	7 (20.0)	
Do you think the test items of the “outcome evaluation” are aligned with the evaluation objectives?						0.562
Examinees (n=116)	2 (1.7)	5 (4.3)	19 (16.4)	64 (55.2)	26 (22.4)	
Former examinees (n=35)	0	0	9 (25.7)	20 (57.1)	6 (17.1)	

a) The chi-square test was conducted to compare the differences between current test-takers and former test-takers in response results (α=0.05).

**Table 6.** Survey results on the participating professors (test item developers and test examiners) and dental school faculty members’ perceptions of the validity of the clinical skill test’s evaluation objectives

Evaluation objectives	Response (no., %)					P-value^a)^
	Strongly disagree	Disagree	Neutral	Agree	Strongly agree	
1. Patient interview, history taking, diagnosis and treatment planning						0.293
Test item developers (n=16)	1 (6.2)	0	0	7 (43.8)	8 (50.0)	
Test examiners (n=32)	0	0	2 (6.2)	9 (28.1)	21 (65.6)	
Dental school faculty members (n=24)	0	0	3 (12.5)	5 (20.8)	16 (66.7)	
2. Oromaxillofacial cranial nerve examination						0.154
Test item developers (n=16)	3 (18.8)	2 (12.5)	4 (25.0)	3 (18.8)	4 (25.0)	
Test examiners (n=32)	0	5 (15.6)	8 (25.0)	15 (46.9)	4 (12.5)	
Dental school faculty members (n=24)	0	2 (8.3)	7 (29.2)	9 (37.5)	6 (25.0)	
3. Temporomandibular disorder palpation test						0.398
Test item developers (n=16)	1 (6.2)	3 (18.8)	2 (12.5)	5 (31.2)	5 (31.2)	
Test examiners (n=32)	0	2 (6.2)	7 (21.9)	17 (53.1)	6 (18.8)	
Dental school faculty members (n=24)	0	1 (4.2)	4 (16.7)	11 (45.8)	8 (33.3)	
4. Pulp test						0.025^*^
Test item developers (n=16)	1 (6.2)	1 (6.2)	0	6 (37.5)	8 (50.0)	
Test examiners (n=32)	0	0	6 (18.8)	15 (46.9)	11 (34.4)	
Dental school faculty members (n=24)	1 (4.2)	0	3 (12.5)	4 (16.7)	16 (66.7)	
5. Periodontal test						0.157
Test item developers (n=16)	1 (6.2)	1 (6.2)	1 (6.2)	5 (31.2)	8 (50.0)	
Test examiners (n=32)	0	0	3 (9.4)	16 (50.0)	13 (40.6)	
Dental school faculty members (n=24)	0	1 (4.2)	2 (8.3)	5 (20.8)	16 (66.7)	
6. Intraoral X-ray taking						0.429
Test item developers (n=16)	2 (12.5)	1 (6.2)	3 (18.8)	6 (37.5)	4 (25.0)	
Test examiners (n=32)	1 (3.1)	3 (9.4)	7 (21.9)	11 (34.4)	10 (31.2)	
Dental school faculty members (n=24)	0	0	3 (12.5)	9 (37.5)	12 (50.0)	
7. Local anesthesia						0.412
Test item developers (n=16)	1 (6.2)	0	2 (12.5)	4 (25.0)	9 (56.2)	
Test examiners (n=32)	0	0	5 (15.6)	13 (40.6)	14 (43.8)	
Dental school faculty members (n=24)	0	0	2 (8.3)	6 (25.0)	16 (66.7)	
8. Rubber dam application						0.387
Test item developers (n=16)	1 (6.2)	0	0	6 (37.5)	9 (56.2)	
Test examiners (n=32)	0	0	4 (12.5)	15 (46.9)	13 (40.6)	
Dental school faculty members (n=24)	0	0	3 (12.5)	8 (33.3)	13 (54.2)	
9. Topical fluoride application						0.431
Test item developers (n=16)	1 (6.2)	3 (18.8)	4 (25.0)	4 (25.0)	4 (25.0)	
Test examiners (n=32)	0	3 (9.4)	6 (18.8)	15 (46.9)	8 (25.0)	
Dental school faculty members (n=24)	0	1 (4.2)	5 (20.8)	8 (33.3)	10 (41.7)	
10. Pit and fissure sealant						0.784
Test item developers (n=16)	1 (6.2)	1 (6.2)	3 (18.8)	4 (25.0)	7 (43.8)	
Test examiners (n=32)	0	1 (3.1)	5 (15.6)	15 (46.9)	11 (34.4)	
Dental school faculty members (n=24)	0	1 (4.2)	5 (20.8)	8 (33.3)	10 (41.7)	
11. Scaling						0.611
Test item developers (n=16)	1 (6.2)	1 (6.2)	1 (6.2)	6 (37.5)	7 (43.8)	
Test examiners (n=32)	0	2 (6.2)	3 (9.4)	15 (46.9)	12 (37.5)	
Dental school faculty members (n=24)	0	0	2 (8.3)	8 (33.3)	14 (58.3)	
12. Root planning						0.289
Test item developers (n=16)	1 (6.2)	2 (12.5)	0	5 (31.2)	8 (50.0)	
Test examiners (n=32)	0	1 (3.1)	4 (12.5)	16 (50.0)	11 (34.4)	
Dental school faculty members (n=24)	0	1 (4.2)	2 (8.3)	8 (33.3)	13 (54.2)	
13. Simple extraction						0.455
Test item developers (n=16)	1 (6.2)	2 (12.5)	1 (6.2)	5 (31.2)	7 (43.8)	
Test examiners (n=32)	0	1 (3.1)	3 (9.4)	12 (37.5)	16 (50.0)	
Dental school faculty members (n=24)	0	0	3 (12.5)	6 (25.0)	15 (62.5)	
14. Suturing skills						0.631
Test item developers (n=16)	1 (6.2)	0	0	7 (43.8)	8 (50.0)	
Test examiners (n=32)	0	0	3 (9.4)	14 (43.8)	15 (46.9)	
Dental school faculty members (n=24)	0	1 (4.2)	2 (8.3)	8 (33.3)	13 (54.2)	
15. Space management for primary and mixed dentition						0.039^*^
Test item developers (n=16)	4 (25.0)	2 (12.5)	3 (18.8)	6 (37.5)	1 (6.2)	
Test examiners (n=32)	0	2 (6.2)	16 (50.0)	10 (31.2)	4 (12.5)	
Dental school faculty members (n=24)	2 (8.3)	2 (8.3)	6 (25.0)	7 (29.2)	7 (29.2)	
16. Oral hygiene education						0.271
Test item developers (n=16)	2 (12.5)	1 (6.2)	2 (12.5)	7 (43.8)	4 (25.0)	
Test examiners (n=32)	1 (3.1)	0	4 (12.5)	16 (50.0)	11 (34.4)	
Dental school faculty members (n=24)	0	1 (4.2)	3 (12.5)	7 (29.2)	13 (54.2)	
17. Endodontic treatment						0.150
Test item developers (n=16)	1 (6.2)	0	2 (12.5)	4 (25.0)	9 (56.2)	
Test examiners (n=32)	0	1 (3.1)	3 (9.4)	18 (56.2)	10 (31.2)	
Dental school faculty members (n=24)	0	1 (4.2)	1 (4.2)	8 (33.3)	14 (58.3)	
18. Amalgam restoration in posterior teeth						0.192
Test item developers (n=16)	1 (6.2)	3 (18.8)	0	4 (25.0)	8 (50.0)	
Test examiners (n=32)	0	2 (6.2)	8 (25.0)	11 (34.4)	11 (34.4)	
Dental school faculty members (n=24)	0	2 (8.3)	6 (25.0)	8 (33.3)	8 (33.3)	
19. Resin composite restoration in anterior teeth						0.351
Test item developers (n=16)	1 (6.2)	1 (6.2)	1 (6.2)	4 (25.0)	9 (56.2)	
Test examiners (n=32)	0	1 (3.1)	5 (15.6)	15 (46.9)	11 (34.4)	
Dental school faculty members (n=24)	0	2 (8.3)	6 (25.0)	8 (33.3)	8 (33.3)	
20. Gold inlay cavity preparation in posterior teeth						0.458
Test item developers (n=16)	1 (6.2)	0	1 (6.2)	5 (31.2)	9 (56.2)	
Test examiners (n=32)	0	0	5 (15.6)	15 (46.9)	12 (37.5)	
Dental school faculty members (n=24)	2 (8.3)	1 (4.2)	3 (12.5)	7 (29.2)	11 (45.8)	
21. Gold crown preparation in posterior teeth and fabrication of a temporary crown						0.525
Test item developers (n=16)	1 (6.2)	0	1 (6.2)	4 (25.0)	10 (62.5)	
Test examiners (n=32)	0	0	4 (12.5)	13 (40.6)	15 (46.9)	
Dental school faculty members (n=24)	0	0	1 (4.2)	10 (41.7)	13 (54.2)	
22. Porcelain fused to metal crown in anterior teeth and fabrication of a temporary crown						0.449
Test item developers (n=16)	1 (6.2)	0	2 (12.5)	3 (18.8)	10 (62.5)	
Test examiners (n=32)	0	0	5 (15.6)	13 (40.6)	14 (43.8)	
Dental school faculty members (n=24)	0	1 (4.2)	2 (8.3)	8 (33.3)	13 (54.2)	

* P<0.05.

a) The chi-squared test was conducted to compare the differences between the groups in response results (α=0.05).

**Table 7.** Survey results on the participating professors’ (test item developers and test examiners) perceptions of the overlap between the clinical skill test's evaluation objectives and dental specialty areas

Evaluation objectives	Response (no., %)					P-value^a)^
	Strongly agree	Agree	Neutral	Disagree	Strongly disagree	
1. patient interview, history taking, diagnosis, and treatment planning						0.905
Test item developers (n=16)	0	0	4 (25.0)	3 (18.8)	9 (56.2)	
Test examiners (n=32)	1 (3.1)	2 (6.2)	7 (21.9)	8 (25.0)	14 (43.8)	
2. Oromaxillofacial cranial nerve examination						0.137
Test item developers (n=16)	5 (31.3)	2 (12.5)	3 (18.8)	2 (12.5)	3 (18.8)	
Test examiners (n=32)	3 (9.4)	8 (25.0)	10 (31.2)	9 (28.1)	2 (6.2)	
3. Temporomandibular disorder palpation test						0.307
Test item developers (n=16)	2 (12.5)	4 (25.0)	5 (31.2)	2 (12.5)	3 (18.8)	
Test examiners (n=32)	3 (9.4)	4 (12.5)	12 (37.5)	11 (34.4)	2 (6.2)	
4. Pulp test						0.534
Test item developers (n=16)	0	1 (6.2)	4 (25.0)	2 (12.5)	9 (56.2)	
Test examiners (n=32)	0	2 (6.2)	11 (34.4)	8 (25.0)	11 (34.4)	
5. Periodontal test						0.571
Test item developers (n=16)	0	1 (6.2)	4 (25.0)	2 (12.5)	9 (56.2)	
Test examiners (n=32)	1 (3.1)	1 (3.1)	12 (37.5)	7 (21.9)	11 (34.4)	
6. Intraoral X-ray taking						0.432
Test item developers (n=16)	1 (6.2)	0	4 (25.0)	2 (12.5)	9 (56.2)	
Test examiners (n=32)	1 (3.1)	2 (6.2)	8 (25.0)	10 (31.2)	11 (34.4)	
7. Local anesthesia						0.478
Test item developers (n=16)	1 (6.2)	0	3 (18.8)	3 (18.8)	9 (56.2)	
Test examiners (n=32)	1 (3.1)	1 (3.1)	7 (21.9)	12 (37.5)	11 (34.4)	
8. Rubber dam application						0.413
Test item developers (n=16)	1 (6.2)	0	4 (25.0)	2 (12.5)	9 (56.2)	
Test examiners (n=32)	1 (3.1)	2 (6.2)	6 (18.8)	11 (34.4)	12 (37.5)	
9. Topical fluoride application						0.494
Test item developers (n=16)	1 (6.2)	0	3 (18.8)	4 (25.0)	8 (50.0)	
Test examiners (n=32)	1 (3.1)	0	8 (25.0)	13 (40.6)	10 (31.2)	
10. Pit and fissure sealant						0.785
Test item developers (n=16)	1 (6.2)	0	3 (18.8)	4 (25.0)	8 (50.0)	
Test examiners (n=32)	1 (3.1)	1 (3.1)	7 (21.9)	12 (37.5)	11 (34.4)	
11. Scaling						0.673
Test item developers (n=16)	1 (6.2)	0	3 (18.8)	3 (18.8)	9 (56.2)	
Test examiners (n=32)	1 (3.1)	2 (6.2)	6 (18.8)	10 (31.2)	13 (40.6)	
12. Root planning						0.413
Test item developers (n=16)	2 (12.5)	3 (18.8)	3 (18.8)	8 (50.0)	0	
Test examiners (n=32)	1 (3.1)	3 (9.4)	7 (21.9)	10 (31.2)	11 (34.4)	
13. Simple extraction						0.575
Test item developers (n=16)	1 (6.2)	0	4 (25.0)	2 (12.5)	9 (56.2)	
Test examiners (n=32)	1 (3.1)	3 (9.4)	7 (21.9)	10 (31.2)	11 (34.4)	
14. Suturing skills						0.819
Test item developers (n=16)	0	0	4 (25.0)	3 (18.8)	9 (56.2)	
Test examiners (n=32)	1 (3.1)	0	9 (28.1)	9 (28.1)	13 (40.6)	
15. Space management for primary and mixed dentition						0.682
Test item developers (n=16)	2 (12.5)	3 (18.8)	7 (43.8)	3 (18.8)	1 (6.2)	
Test examiners (n=32)	3 (9.4)	3 (9.4)	12 (37.5)	12 (37.5)	2 (6.2)	
16. Oral hygiene education						0.813
Test item developers (n=16)	1 (6.2)	0	2 (12.5)	5 (31.2)	8 (50.0)	
Test examiners (n=32)	1 (3.1)	1 (3.1)	8 (25.0)	9 (28.1)	13 (40.6)	
17. Endodontic treatment						0.423
Test item developers (n=16)	1 (6.2)	1 (6.2)	3 (18.8)	3 (18.8)	8 (50.0)	
Test examiners (n=32)	1 (3.1)	3 (9.4)	8 (25.0)	12 (37.5)	8 (25.0)	
18. Amalgam restoration in posterior teeth						0.645
Test item developers (n=16)	1 (6.2)	0	5 (31.2)	2 (12.5)	8 (50.0)	
Test examiners (n=32)	1 (3.1)	2 (6.2)	10 (31.2)	8 (25.0)	11 (34.4)	
19. Resin composite restoration in anterior teeth						0.668
Test item developers (n=16)	1 (6.2)	0	3 (18.8)	4 (25.0)	8 (50.0)	
Test examiners (n=32)	1 (3.1)	2 (6.2)	10 (31.2)	8 (25.0)	11 (34.4)	
20. Gold inlay cavity preparation in posterior teeth						0.444
Test item developers (n=16)	1 (6.2)	0	3 (18.8)	3 (18.8)	9 (56.2)	
Test examiners (n=32)	1 (3.1)	1 (3.1)	10 (31.2)	10 (31.2)	10 (31.2)	
21. Gold crown preparation in posterior teeth and fabrication of a temporary crown						0.417
Test item developers (n=16)	1 (6.2)	0	3 (18.8)	3 (18.8)	9 (56.2)	
Test examiners (n=32)	1 (3.1)	1 (3.1)	11 (34.4)	9 (28.1)	10 (31.2)	
22. Porcelain fused to metal crown in anterior teeth and fabrication of a temporary crown						0.294
Test item developers (n=16)	1 (6.2)	0	3 (18.8)	3 (18.8)	9 (56.2)	
Test examiners (n=32)	1 (3.1)	1 (3.1)	11 (34.4)	10 (31.2)	9 (28.1)	

a) The chi-squared test was conducted to compare the differences between the groups in response results (α=0.05).

## Footnotes

**Data availability**
Data files are available from Harvard Dataverse: https://doi.org/10.7910/DVN/XSCXDR Dataset 1. Response data from the survey of current and former examinees regarding the evaluation objectives of the clinical skill test (Table 5).

Dataset 2. Response data from the survey of the participating professors and dental school faculty members regarding their perceptions of the validity of the clinical skill test’s evaluation objectives (Table 6).

Dataset 3. Response data from the survey of the participating professors regarding their perceptions of the overlap between the clinical skill test’s evaluation objectives and dental specialty areas (Table 7).

## Supplementary materials

Supplementary files are available from Harvard Dataverse: https://doi.org/10.7910/DVN/XSCXDR

Supplement 1. National-level dental practitioner competencies in South Korea.

Supplement 2. Survey questionnaire for current and former examinees on the evaluation objectives of the clinical skill test.

Supplement 3. Survey questionnaire for professors participating in the examination on the validity of the clinical skill test’s evaluation objectives and the extent to which they overlap with dental specialty areas.

Supplement 4. Survey questionnaire for dental school faculty members on the validity of the clinical skill test’s evaluation objectives.

Supplement 5. Audio recording of the abstract.
