# Supplementary material for: Revised evaluation objectives of the Korean Dentist Clinical Skill Test: a survey study and focus group interviews
Source: J Educ Eval Health Prof. 2024 May 30;21:11. doi: 10.3352/jeehp.2024.21.11 (PMC11219220; doi:10.3352/jeehp.2024.21.11)
Supplement: Supplementary file 5 — Supplement 2. Survey questionnaire for current and former examinees on the evaluation objectives of the clinical skill test. [file jeehp-21-11-suppl2.docx]

This survey is being conducted for the study “Revised evaluation objectives of the Korean Dentist Clinical Skill Test.” We kindly request your feedback for the improvement of the national dental licensing examination. Your responses to this survey will be used solely for research purposes.

1. Do you know the evaluation objectives for the Korean Dentist Clinical Skill Test?

① Strongly disagree ② Disagree ③ Neutral ④ Agree ⑤ Strongly agree

2. Have you taken any preparation training for the examination offered by your school?

① Strongly disagree ② Disagree ③ Neutral ④ Agree ⑤ Strongly agree

3. Did the evaluation objectives help you prepare for the examination?

① Strongly disagree ② Disagree ③ Neutral ④ Agree ⑤ Strongly agree

4. Did the evaluation objectives help you develop basic competencies as a dentist?

① Strongly disagree ② Disagree ③ Neutral ④ Agree ⑤ Strongly agree

5. Do you think the “process evaluation” objectives reflect the competencies required of new dentists?

① Strongly disagree ② Disagree ③ Neutral ④ Agree ⑤ Strongly agree

6. Do you think the “process evaluation” objectives reflect the educational content of your school?

① Strongly disagree ② Disagree ③ Neutral ④ Agree ⑤ Strongly agree

7. Do you think the test items of the “process evaluation” are aligned with the evaluation objectives?

① Strongly disagree ② Disagree ③ Neutral ④ Agree ⑤ Strongly agree

8. Do you think the “outcome evaluation” objectives reflect the competencies required of new dentists?

① Strongly disagree ② Disagree ③ Neutral ④ Agree ⑤ Strongly agree

9. Do you think the “outcome evaluation” objectives reflect the educational content of your school?

① Strongly disagree ② Disagree ③ Neutral ④ Agree ⑤ Strongly agree

10. Do you think the test items of the “outcome evaluation” are aligned with the evaluation objectives?

① Strongly disagree ② Disagree ③ Neutral ④ Agree ⑤ Strongly agree
