# Supplementary material for: Revised evaluation objectives of the Korean Dentist Clinical Skill Test: a survey study and focus group interviews
Source: J Educ Eval Health Prof. 2024 May 30;21:11. doi: 10.3352/jeehp.2024.21.11 (PMC11219220; doi:10.3352/jeehp.2024.21.11)
Supplement: Supplementary file 7 — Supplement 4. Survey questionnaire for dental school faculty members on the validity of the clinical skill test’s evaluation objectives. [file jeehp-21-11-suppl4.docx]

This survey is being conducted for the study “Revised evaluation objectives of the Korean Dentist Clinical Skill Test.” We kindly request your feedback for the improvement of the national dental licensing examination. Your responses to this survey will be used solely for research purposes.

The following are the evaluation objectives for the Korean Dentist Clinical Skill Test as announced by the Korean Health Personnel Licensing Examination Institute.

| Evaluation objectives (22 items) |
| --- |
| 1. Patient interview, history taking, diagnosis, and treatment planning  2. Oromaxillofacial cranial nerve examination  3. Temporomadibular disorder palpation test  4. Pulp test  5. Periodontal test  6. Intraoral X-ray taking  7. Local anesthesia  8. Rubber dam application  9. Topical fluoride application  10. Pit and fissure sealant  11. Scaling  12. Root planning  13. Simpe extraction  14. Suturing skills  15. Space management for primary and mixed dentition  16. Oral hygiene education  17. Endodontic treatment  18. Amalgam restoration in posterior teeth  19. Resin composite restoration in anterior teeth  20. Gold inlay cavity preparation in posterior teeth  21. Gold crown preparation in posterior teeth and fabrication of temporary crown  22. Porcelain fused to metal crown in anterior teeth and fabrication of temporary crown |

**1. Is it appropriate to assess the evaluation objectives through the clinical skill test?**

1-1. Patient interview, history taking, diagnosis and treatment planning

① Strongly disagree ② Disagree ③ Neutral ④ Agree ⑤ Strongly agree

1-2. Oromaxillofacial cranial nerve examination

① Strongly disagree ② Disagree ③ Neutral ④ Agree ⑤ Strongly agree

1-3. Temporomadibular disorder palpation test

① Strongly disagree ② Disagree ③ Neutral ④ Agree ⑤ Strongly agree

1-4. Pulp test

① Strongly disagree ② Disagree ③ Neutral ④ Agree ⑤ Strongly agree

1-5. Periodontal test

① Strongly disagree ② Disagree ③ Neutral ④ Agree ⑤ Strongly agree

1-6. Intraoral X-ray taking

① Strongly disagree ② Disagree ③ Neutral ④ Agree ⑤ Strongly agree

1-7. Local anesthesia

① Strongly disagree ② Disagree ③ Neutral ④ Agree ⑤ Strongly agree

1-8. Rubber dam application

① Strongly disagree ② Disagree ③ Neutral ④ Agree ⑤ Strongly agree

1-9. Topical fluoride application

① Strongly disagree ② Disagree ③ Neutral ④ Agree ⑤ Strongly agree

1-10. Pit and fissure sealant

① Strongly disagree ② Disagree ③ Neutral ④ Agree ⑤ Strongly agree

1-11. Scaling

① Strongly disagree ② Disagree ③ Neutral ④ Agree ⑤ Strongly agree

1-12. Root planning

① Strongly disagree ② Disagree ③ Neutral ④ Agree ⑤ Strongly agree

1-13. Simpe extraction

① Strongly disagree ② Disagree ③ Neutral ④ Agree ⑤ Strongly agree

1-14. Suturing skills

① Strongly disagree ② Disagree ③ Neutral ④ Agree ⑤ Strongly agree

1-15. Space management for primary and mixed dentition

① Strongly disagree ② Disagree ③ Neutral ④ Agree ⑤ Strongly agree

1-16. Oral hygiene education

① Strongly disagree ② Disagree ③ Neutral ④ Agree ⑤ Strongly agree

1-17. Endodontic treatment

① Strongly disagree ② Disagree ③ Neutral ④ Agree ⑤ Strongly agree

1-18. Amalgam restoration in posterior teeth

① Strongly disagree ② Disagree ③ Neutral ④ Agree ⑤ Strongly agree

1-19. Resin composite restoration in anterior teeth

① Strongly disagree ② Disagree ③ Neutral ④ Agree ⑤ Strongly agree

1-20. Gold inlay cavity preparation in posterior teeth

① Strongly disagree ② Disagree ③ Neutral ④ Agree ⑤ Strongly agree

1-21. Gold crown preparation in posterior teeth and fabrication of temporary crown

① Strongly disagree ② Disagree ③ Neutral ④ Agree ⑤ Strongly agree

1-22. Porcelain fused to metal crown in anterior teeth and fabrication of temporary crown

① Strongly disagree ② Disagree ③ Neutral ④ Agree ⑤ Strongly agree
